# Percutaneous Retrieval of a Micra™ Leadless Pacemaker Using a One-tine–based Snaring Technique: The Tine Is Durable but Maybe It Should Be the Last Resort

**DOI:** 10.19102/icrm.2026.17054

**Published:** 2026-05-15

**Authors:** Mohamed Shokr

**Affiliations:** 1 Cardiac Electrophysiology Department, Northern Light Cardiology/Eastern Maine Medical Center, Bangor, ME, USA

**Keywords:** Dislodgment, Micra leadless pacemaker, retrieval, snaring, tine-based snaring

## Abstract

Micra™ retrieval techniques involve snaring the proximal knob; however, device orientation can hinder knob access. Unorthodox retrieval methods included a two-snare approach and a “snare-in-snare” technique. We describe a single-tine–based snaring approach of a Micra™ leadless pacemaker (Medtronic, Minneapolis, MN, USA) 4 days after implantation due to loss of capture. An 89-year-old man with a history of chronic kidney disease, hypertension, and transcatheter aortic valve replacement complicated by complete heart block underwent Micra™ implantation. Four days later, he presented with syncope. Interrogation showed an elevated capture threshold with intermittent loss of capture. Chest radiography confirmed an upside-down orientation in the right ventricular outflow tract. A 27-Fr outer-diameter Aveir™ Introducer Sheath (Abbott, Chicago, IL, USA) was advanced via the right femoral vein. The Aveir™ leadless pacemaker (Abbott) was implanted in a lower septal position. Multiple attempts to snare the Micra™ device’s retrieval knob using the Aveir™ Retrieval Catheter (Abbott) and a Goose Neck Snare (20-mm loop diameter, 102 cm; Covidien [Medtronic], Dublin, Ireland) through a steerable sheath failed. A figure-of-eight stitch was placed around the introducer sheath and left untied. The snare engaged a partially free tine. Gentle traction confirmed secure engagement, and controlled traction disengaged the remaining tines. The device was withdrawn into the inferior vena cava but could not be pulled into the sheath due to angulation. The entire system was removed, and the groin stitch was tied. However, the Micra™ dislodged into the groin subcutaneous tissue. Iliofemoral angiography via internal jugular vein access confirmed no extravasation, and the device was explanted through a small groin incision using forceps. Percutaneous retrieval of a Micra™ leadless pacemaker, with short dwell time, is feasible using a one-tine–based snaring technique when snaring of the proximal retrieval knob fails. The tine is durable; however, caution should be exercised.

## Introduction

The Micra™ Transcatheter Pacing System (TPS) (Medtronic, Minneapolis, MN, USA) is a leadless pacemaker designed for single-chamber ventricular pacing, anchored to the right ventricular myocardium using four nitinol tines. Early post-implantation complications, such as elevated pacing thresholds due to micro-dislodgment or tissue reaction, may necessitate device retrieval.^1^ Standard retrieval techniques involve snaring the proximal retrieval knob; however, device orientation can hinder knob access.^2^ We describe a novel single-tine–based snaring technique for Micra™ retrieval in a patient with loss of capture shortly after implantation, offering a safe and effective alternative approach.

## Case presentation

**Figure 1:**
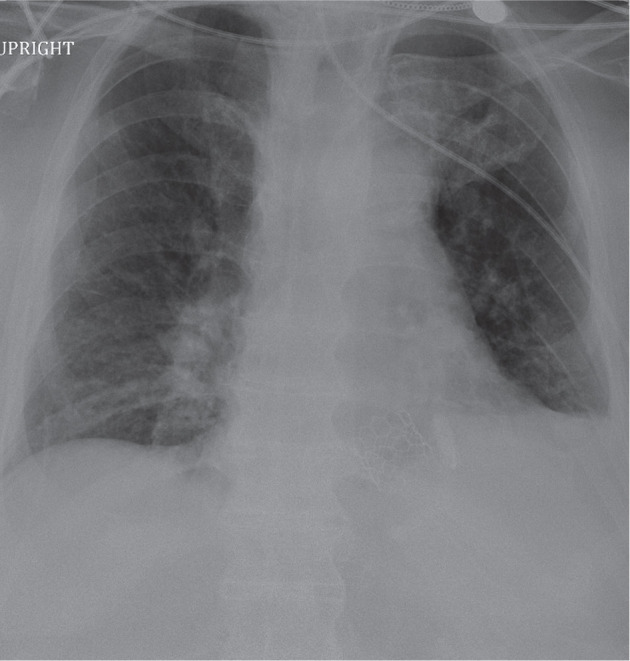
Chest radiograph showing the vertical orientation of the Micra™ upside down in the right ventricular outflow tract.

An 89-year-old man with chronic kidney disease, diabetes mellitus, hypertension, obstructive sleep apnea, bifascicular block, first-degree atrioventricular block, and recent transcatheter aortic valve replacement complicated by complete heart block underwent Micra™ TPS implantation 4 days prior by another operator. The initial procedure, performed via right femoral vein access, was uncomplicated, with a capture threshold of 0.38 V at 0.24 ms, an impedance of 420 Ω, and an R-wave sensing of 13.5 mV. Two tines were confirmed to be engaged via pull-and-hold testing. Four days later, the patient presented with recurrent syncope due to trauma and intermittent loss of capture on an electrocardiogram, revealing underlying complete heart block. Device interrogation showed an elevated capture threshold of 4.13 V at 0.24 ms, with impedance of 380 Ω and sensing of 9.1 mV. Chest radiography confirmed an upside-down device orientation in the right ventricular outflow tract (RVOT) without gross dislodgment **(Figure 1)**, suggesting micro-dislodgment or early tissue reaction at the electrode–myocardium interface.

**Figure 2:**
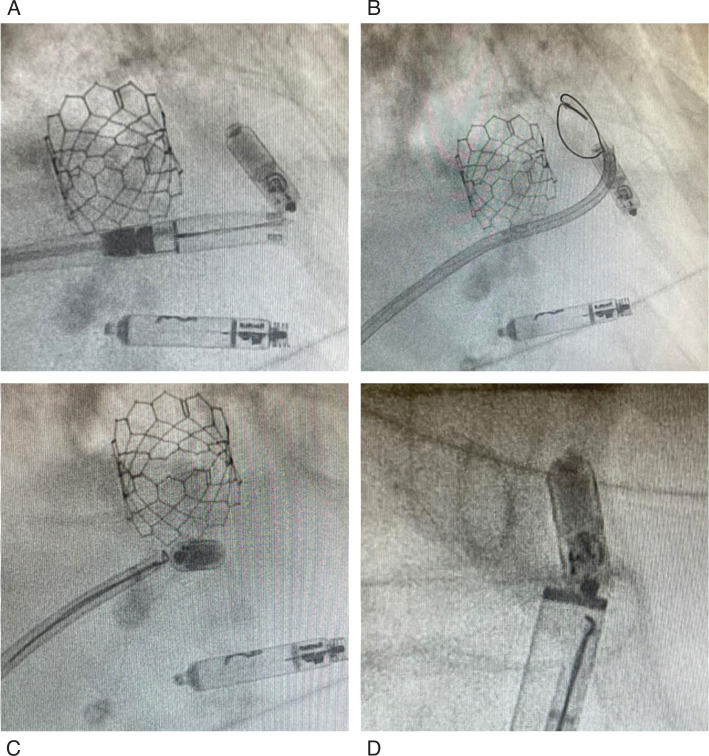
**A:** The Aveir™ retrieval tool catching on the free tine (right anterior oblique). **B:** A medium-curl Agilis™ and a 20-mm Goose Neck Snare in the right ventricular outflow tract above the Micra™ in a failed trial to snare the retrieval knob (right anterior oblique). **C:** The Goose Neck Snare captured the free tine of the Micra™ (left anterior oblique). **D:** The Micra™ failed to be pulled inside the introducer sheath. Note the snare capturing the tine only.

After informed consent was obtained, the decision was made to retrieve the Micra™ device and implant an Aveir™ leadless pacemaker (Abbott, Chicago, IL, USA) using the Aveir™ Retrieval Catheter (LSCR111; Abbott). Under general anesthesia, a 25-Fr inner-and 27-Fr outer-diameter Aveir™ Introducer Sheath (Abbott) was advanced via the right femoral vein. Due to the absence of an underlying escape rhythm, we proceeded with the implant process first. The Aveir™ leadless pacemaker was implanted uneventfully in a lower septal position with adequate pacing and sensing parameters. Multiple attempts to snare the Micra’s proximal retrieval knob using the Aveir™ Retrieval Catheter failed due to the device’s orientation in the RVOT, which prevented its advancement closer to the pulmonary valve **(Figure 2A)**. Instead, the snare frequently caught on a nitinol tine by chance. The retrieval catheter was removed, and a medium-curl steerable sheath (Agilis™ NxT; Abbott) was introduced through the sheath. An Amplatz Goose Neck Snare (20-mm loop diameter, 120-mm snare length, 6 Fr, 102 cm; Covidien [Medtronic], Dublin, Ireland) was advanced through the steerable sheath in another trial to snare the knob.

**Figure 3:**
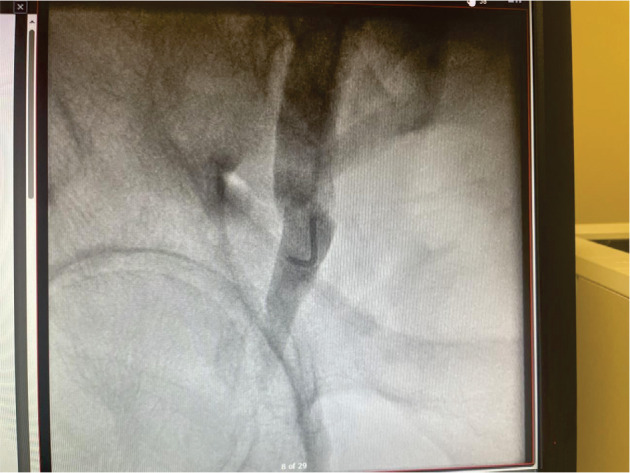
Venogram through internal jugular vein access shows intact veins without extravasation.

**Figure 4:**
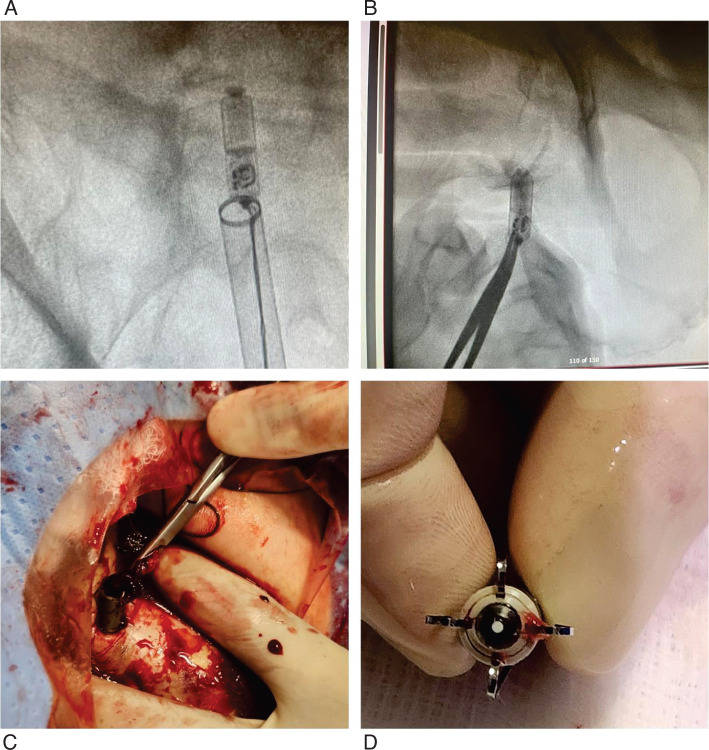
**A:** The whole system is at the groin while being pulled off the body. **B, C:** The Micra™ device, dislodged under the skin, is being captured by a forceps under fluoroscopic guidance. **D**: The device outside the body with intact tines.

Attempts to encircle the retrieval knob were unsuccessful due to RVOT orientation **(Figure 2B)**. Instead, the snare engaged a partially free distal nitinol tine, and we thought to use this to disengage the whole device. We elected to secure the groin access first in case we needed to remove the whole system including the introducer sheath at once. A figure-of-eight stitch was placed around the introducer sheath and left untied. Gentle traction confirmed secure engagement of the tine, and, with back-support from the steerable sheath, controlled traction disengaged the remaining tines. The device was withdrawn into the inferior vena cava but could not be pulled into the introducer sheath due to angulation. The entire system was removed, and the groin stitch was tied. However, the Micra™ dislodged into the groin subcutaneous tissue, appearing stable under fluoroscopy. Iliofemoral angiography via internal jugular vein access confirmed no extravasation, tears, or intravascular dislodgment **(Figure 3)**. Vascular surgery was consulted; however, no vascular intervention was needed, as the device was explanted through a small groin incision using forceps **(Figures 4B and 4C)**, under fluoroscopic guidance. All tines were intact **(Figure 4D)**. Immediate postoperative echocardiography showed no effusion or trivial tricuspid regurgitation. The total procedure time was 48 min. The patient was monitored overnight and discharged without complications.

## Discussion

This case demonstrates the successful retrieval of a Micra™ TPS 4 days post-implantation due to elevated pacing thresholds causing loss of capture and recurrent syncope. Snaring a single nitinol tine suggests partial dislodgment as the likely cause of the rising thresholds, consistent with early post-implantation complications.^1^ Standard Micra™ retrieval involves snaring the proximal retrieval knob using a 7-mm snare through the delivery catheter, a larger snare via a steerable sheath, or the Aveir™ Retrieval Catheter. Early encapsulation is uncommon but may occur in patients with heightened inflammatory responses, complicating retrieval. The novel single-tine–based snaring technique described here leverages an unattached nitinol tine’s accessibility. This approach requires operator expertise in snaring techniques and carries a risk of tine fracture, which could lead to device dislodgment. The steerable sheath’s lack of counter-traction may increase the risk of myocardial avulsion, though this is less likely with recently implanted devices due to minimal tissue ingrowth. Previous unorthodox retrieval methods include a two-snare approach, capturing the Micra™ body and one end, and a “snare-in-snare” technique.^3^ Both techniques are safer compared to snaring a single tine.

Taborsky et al. reported retrieving a dislodged Micra™ in a patient with infective endocarditis; one figure suggests a single-loop retrieval snare (Cook Medical, Bloomington, IN, USA) captured all four tines via a steerable sheath (Agilis™ NxT) through the right femoral vein.^4^ Kojima et al. described a single snare pushing technique to reorient a Micra™ fixed in the tricuspid valve annulus using a multiple-loop snare (4–8 mm, EN Snare; Merit Medical Systems, South Jordan, UT, USA) via the right internal jugular vein. The tine was snared; however, tethers remained attached during repositioning.^5^ In contrast, we targeted a single tine to retrieve a fully implanted device 4 days post-implantation. Bonner et al. demonstrated retrieval of a 3-year-old Micra™ device from a cadaver using a maximum force of 1.9 pounds, suggesting feasibility in longer dwell times with minimal force.^6^

The nitinol tines are designed with an approximate safety factor of 26× with respect to tearing tissue when pulling out the tines. It is more likely for the tines to flex and unfold rather than tear the tissue.^7^

The nitinol tines are made of superelastic nickel–titanium alloy for fixation within the right ventricular myocardium. While exact proprietary tensile strength values remain undisclosed in Medtronic specifications, superelastic nitinol in comparable medical implants generally exhibits ultimate tensile strengths of 800–1500 MPa, supporting high fatigue resistance, elastic recovery under cyclic cardiac loads, and reliable long-term performance. Post-market surveillance via the MAUDE database has identified rare tine fractures.^8^ Our case provides proof that a tine can withstand the snaring force that was enough to extract the remaining tines from the myocardium and pull the device to the groin area.

This case highlights the feasibility of single-tine–based snaring when retrieval knob capture is not possible. The technique’s applicability for devices with longer dwell times remains uncertain, as encapsulation may hinder tine engagement, and the force applied might not be enough to extract the encapsulated device from the myocardium. Preparation for groin access management is critical, as withdrawing the entire system, including the introducer sheath, might need to be done quickly during the case to avoid vascular dislodgment. Although snaring the tine should be considered a last resort, our case demonstrates that it can be employed when other snaring techniques are not feasible.

## Conclusion

Percutaneous retrieval of a Micra™ TPS, with short dwell time, is feasible using a one-tine–based snaring technique when snaring of the proximal retrieval knob fails. As leadless pacing expands, tine-based snaring may enhance the procedural toolkit for retrieval of this device; however, caution should be exercised.
